# A new method for estimating HIV incidence from a single cross-sectional survey

**DOI:** 10.1371/journal.pone.0237221

**Published:** 2020-08-12

**Authors:** Ian E. Fellows, Ray W. Shiraishi, Peter Cherutich, Thomas Achia, Peter W. Young, Andrea A. Kim

**Affiliations:** 1 Fellows Statistics, San Diego, CA, United States of America; 2 Division of Global HIV and TB, U.S. Centers for Disease Control and Prevention, Atlanta, GA, United States of America; 3 National AIDS and STD Control Programme, Ministry of Health, Nairobi, Kenya; 4 Division of Global HIV and TB, U.S. Centers for Disease Control and Prevention, Nairobi, Kenya; London School of Hygiene and Tropical Medicine, UNITED KINGDOM

## Abstract

Estimating incidence from cross-sectional data sources is both important to the understanding of the HIV epidemic and challenging from a methodological standpoint. We develop a new incidence estimator that measures the size of the undiagnosed population and the amount of time spent undiagnosed in order to infer incidence and transmission rates. The estimator is calculated using commonly collected information on testing history and HIV status and, thus, can be deployed in many HIV surveys without additional cost. If ART biomarker status and/or viral load information is available, the estimator can be adjusted for biases in self-reported testing history. The performance of the estimator is explored in two large surveys in Kenya, where we find our point estimates to be consistent with assay-derived estimates, with much smaller standard errors.

## Introduction

Understanding epidemic trends is critical for determining and evaluating strategies for combating HIV. The rate of new HIV infections (incidence) is perhaps the most important indicator for evaluating epidemic control. Unfortunately, while HIV prevalence is comparatively easy to estimate by testing participants in cross-sectional population-based surveys (using highly sensitive and specific serological and molecular tests), incidence is more difficult to measure due to the fact that incident HIV infections share signs and symptoms with many other diseases and therefore often go undiagnosed for significant time periods.

A number of competing methodologies have been developed to estimate disease incidence. If a representative prospective cohort of HIV negative individuals are followed over time, then incidence may be estimated by observing the rate at which new infections occur in the cohort through serial testing for prevalent infection. Prospective cohort studies, while rigorous from a methodological standpoint, are relatively rare due to the high cost of implementation. Additionally, the sample can be rendered non-representative if the initial sample is not selected randomly, subjects drop out of the study in a non-random way, or if subjects change their behavior in response to serial testing.

If two or more cross-sectional surveys are conducted over a number of years, the samples may be treated as a synthetic cohort, and changes in prevalence between surveys may be used to perform inference about incidence [1]. Additionally, country-level and increasingly, sub-national incidence estimates may be inferred from surveillance data using the Spectrum-EPP epidemic models [2, 3]. This approach requires making numerous demographic assumptions regarding migration, fertility and mortality among those with and without HIV infection in the population.

A common method for estimating incidence using a single cross-sectional survey is the immunoassay approach. These assays, based on immunological markers of early HIV disease, are designed to distinguish recent infections from long-term infections. Utilizing the percentage classified as recent by the biomarker, the mean duration of recent infection (MDRI), and the false recency rate (FRR), an incidence estimate may be formulated [4, 5]. MDRI is the average time since infection among recent cases in the population. FRR is the proportion of non-recent infections that would be classified as recent by the assay.

There are two primary limitations of the assay method. First, power and sample size analyses show that the required sample sizes to obtain accurate estimates are quite large (often >10,000 individuals). For general population surveys, this can make incidence estimation prohibitively expensive and for key populations it may be impossible to recruit a sample of sufficient size. Secondly, the calculation depends on the FRR and MDRI, which can vary substantially between populations[6, 7]. The difficulty in estimating these calibration parameters has resulted in most studies not using locally valid parameters, leading to incidence estimates of questionable accuracy. Additionally, the presence of anti-retroviral therapy can lead long-term infections to be classified as recent, inflating the FRR. The effect of this must be accounted for in the FRR value, or if viral load and/or ART biomarkers are available, these may be used to remove treated individuals from those (falsely) classified as recent infections.

In this paper, we describe a new approach to estimating incidence from a cross-sectional sample, including methods for adjusting for errors in self-report. The motivation for this method is based on the cascade of HIV care [8]. The first group in the cascade of care includes infected individuals who have yet to be diagnosed. Since all new HIV infections start out as undiagnosed, the rate that undiagnosed cases are created is the definition of incidence. If the probability that an undiagnosed person transitions to the next step in the cascade (being diagnosed) over a period of time is known, the size of the undiagnosed population is known, and the epidemic is in a stable state during the time period in which individuals in the cross-sectional survey could have been infected, then we can infer incidence. The methodology developed here also allows for calculation of the transmission rate, defined as the expected number of infections per year per infected individual, a metric of considerable public health interest [9]. We applied the method to testing history and biomarker data from two large population-based surveys conducted Kenya in 2007 and 2012.

## Methods

### Mathematical development

Let *H* be an event indicating that an individual in the population is HIV positive at present, *U* indicate that that person has never been diagnosed previously, *N* indicate the population size. Further, let TID be a random variable indicating the time between HIV infection and diagnosis for that person. Throughout we will use the notation *X*^*c*^ to denote the complement of *X* (e.g. *H*^*c*^ represents the event that the individual is not HIV positive).

Suppose that the epidemic is in a steady state such that the rate of infection, which we define as *r*, has been stable for some time and further assume that individuals have been present in the population for longer than any likely time from infection to diagnosis. From Fellows et al. (2015) [10] Eq 1 we have that

$$NP(U)=Nr\int_{0}^{\infty }P(TID>t)dt=NrE(TID),$$

where *NP*(*U*) is the expected number of undiagnosed individuals in the population and *Nr* is the expected number of infections per time unit.

Defining $\lambda =\frac{r}{1-P(H)}$ as incidence (i.e. the rate of infection per time unit among those at risk) and $\tau =\frac{r}{P(H)}$ as transmission rate (i.e. the number of infections per time unit per infected individual) we can rearrange the above as:

$$\begin{array}{l} rE(TID)\;=P(U)\\ \;=P(U|H)P(H)\\ \;r\;=\frac{P(U|H)P(H)}{E(TID)} \end{array}$$

$$\tau =\frac{P(U|H)}{E(TID)}$$ (1)

$$\lambda =\frac{P(U|H)P(H)}{E(TID)(1-P(H))}$$ (2)

The sensitivity of these relations to the steady state assumption is examined in the S1 Appendix.

Eq 1 tells us something interesting from a monitoring standpoint. First, this ratio states that if expected time from infection to diagnosis is constant, lowering the proportion undiagnosed by x% decreases the transmission rate by x%. Second, it states that if we decrease the time from infection to diagnosis by x% this would have to cause a decrease in the proportion undiagnosed by more than x% in order to produce a decrease in the transmission rate. This may be useful in evaluating the effectiveness of interventions where the goal is to increase testing, thereby decreasing the time from infection to diagnosis and hopefully reducing the number of transmissions that would have occurred absent knowing the newly diagnosed individuals’ status.

To estimate *λ* and *τ* we replace the expectations and probabilities in Eq 1 by means ($\hat{E}$) and proportions ($\hat{P}$) from a cross-sectional survey. In the case of more complex sampling designs, these will be weighted means and proportions

$$\hat{\tau }=\frac{\hat{P}(U|H)}{\hat{E}(TID)}$$ (3)

$$\hat{\lambda }=\frac{\hat{P}(U|H)\hat{P}(H)}{\hat{E}(TID)(1-\hat{P}(H))}.$$ (4)

If the (weighted) means and proportions are consistent for their population values, then by Slutsky’s theorem Eqs 3 and 4 are consistent estimators of the transmission and incidence rates. Because HIV status is determined by a laboratory test, we can be confident in its reliability. However, for self-reported survey responses, respondent bias may play a significant role.

### Estimation of *E*(TID)

Time between infection and diagnosis is modeled as the combination of two processes. The first process is regular testing and the second is diagnosis in response to symptom development. These two processes are combined as competing risks in order to estimate *E*(TID).

Cross-sectional HIV surveys often collect self-reported data on whether the study subjects have ever been tested for HIV, and if so, how long ago their last test was. For individuals who engage in HIV testing, if testing behavior is unrelated to HIV infection and they are asymptomatic, infection will have occurred randomly during the time between tests. Similarly, if the timing of the cross-sectional survey is unrelated to testing behavior, the survey will have occurred at a random point between an individual’s last test, and the next test they would have had absent the survey. Thus, the distribution of time since last test at the time of the survey (TLST) is the same as the distribution of time from infection to next regular test among testers. If the testing distribution is uniform across the population, then individuals who are HIV negative may be used as a representative sample in the estimation of the TSLT distribution.

We model time to symptomatic diagnosis as the time from infection to AIDS in the absence of treatment (TAIDS). Brookmeyer and Goedert (1989) modeled this as a Weibull distribution with median 10.052 and mean 10.319 years (scale = 1/0.086, shape = 2.516) [11]. This distribution has been used in a number of incidence estimation methods [12, 13]. Other studies have reported median time to AIDS in non-transfusion populations between 8.2 and 12 years [14].

$$\begin{array}{ll} \hat{E}(TID) & =\int_{0}^{\infty }\hat{P}(TID>t)dt\\ & =\int_{0}^{\infty }\left(\hat{P}(TSLT>t|TESTER)P(TAIDS>t)\hat{P}(TESTER)+P(TAIDS>t)(1-\hat{P}(TESTER))\right)dt \end{array}$$

Where $\hat{P}(TESTER)$ is the sample proportion of individuals reporting having engaged in testing, $\hat{P}(TSLT>t|TESTER)$ is the estimated survival function for time since last test.

For either ease of data collection, or due to respondent unreliability, TSLT is often collected as discrete categories. For instance, a survey may ask whether the last test was 0–6 months ago, 6–12 months ago or >12 months ago. Maximum likelihood inference may be used to estimate the survival function. Let $b_{i}^{-}$ be the lower bound of the interval for tester i, $b_{i}^{+}$ be the upper bound and PW(*X*|*β*_*scale*_,*β*_*shape*_) be the Weibull cumulative distribution function. Then, assuming TSLT follows a Weibull distribution, the likelihood is

$$L(\beta_{scale},\beta_{shape}|b^{+},b^{-})=\prod_{i=1}^{n}(PW(b_{i}^{+}|\beta_{scale},\beta_{shape})-PW(b_{i}^{-}|\beta_{scale},\beta_{shape})),$$

where *n* is the number of testers. Maximizing the above to obtain MLE estimates (${\hat{\beta }}^{MLE}$), which may then be used to construct $\hat{P}(TSLT>t|TESTER)$.

### Estimation of *P*(*U*|*H*)

If subjects are perfectly reliable, then the proportion of undiagnosed cases may be estimated simply as the proportion of HIV positive individuals who report never having received an HIV diagnosis in the past. In some cases, individuals who have been previously diagnosed will claim that they have not been. Suppose *S* is the event that a subject reports a previous diagnosis and *V* is the event of a bio-marker that can identify some subset of individuals who have been previously diagnosed. For example, *V* may indicate an undetectable viral load, which would suggest that the subject is currently on ART and thus must have been diagnosed, or it may indicate a positive test for ART biomarkers in the individual’s bloodstream.

**Fig 1:**
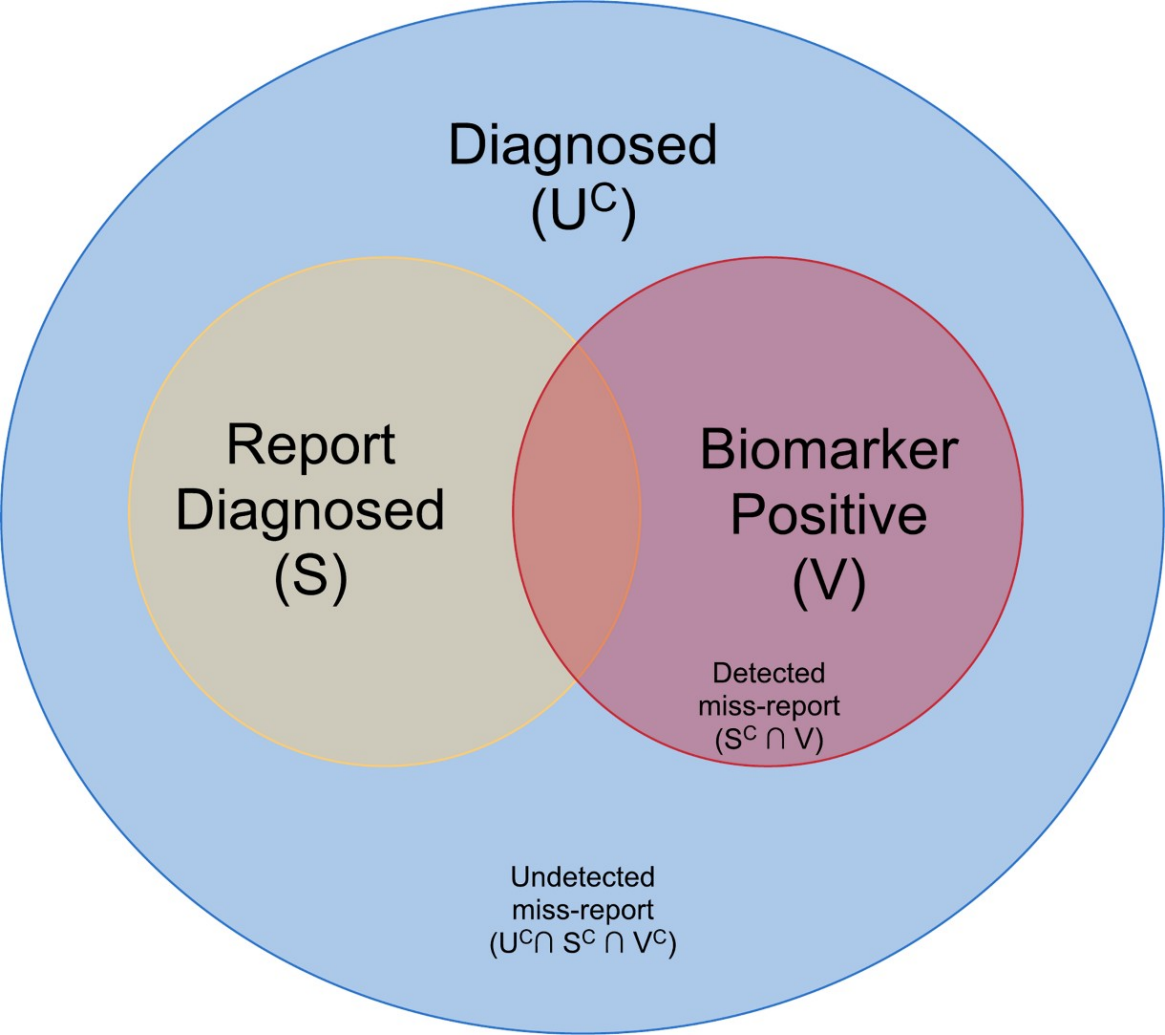
A Venn diagram of the relationship between true undiagnosed status and status as inferred from self-reporting and biomarker positivity.

Fig 1 displays the relationship of *S*, *V* and *U* under the assumption that undiagnosed individuals do not report that they have tested positive in the past. We can express the probability of being undiagnosed in terms of

$$\begin{array}{l} P(S\cup V|H)\;=P(S\cup V|U^{c})(1-P(U|H))\\ \;P(U|H)\;=1-\frac{P(S\cup V|H)}{P(S\cup V|U^{c})}. \end{array}$$

Further assuming that *S* and *V* are independent we have

$$P(S\cup V|U^{c})=P(S|U^{c})+P(V|U^{c})(1-P(S|U^{c})).$$

If *S* and *V* are independent then those with a positive bio-marker report their true status at the same rate as the rest of the diagnosed population, so *P*(*S*|U^*C*^) may be estimated as the proportion of bio-marker positive individuals who report having been previously diagnosed. Independence also implies that *P*(*V*|U^c^) may be estimated as the proportion of individuals who report being HIV positive that have a positive bio-marker.

Combining the previous two equations results in an undiagnosed rate of

$$P(U|H)=1-\frac{P(S\cup V|H)}{P(S|U^{c})+P(V|U^{c})(1-P(S|U^{c}))}.$$

Substituting the sample proportion ($\hat{P}$) in for the probabilities, *P*(*V*|U^c^) with $\hat{P}(V|S)$, and *P*(*S*|U^c^) with $\hat{P}(S|V)$ gives us an estimator for the proportion of undiagnosed positives using only observable quantities

$$\hat{P}(U|H)=1-\frac{\hat{P}(S\cup V|H)}{\hat{P}(S|V)+\hat{P}(V|S)(1-\hat{P}(S|V))}.$$ (5)

The above estimate relies on the independence of *S* and *V*. If this does not hold, we might expect untreated individuals to miss-report at a higher rate than treated individuals due to the treated individuals having higher contact with health providers and taking regular steps to treat their condition. Under these conditions the estimator would only partially correct for the underreporting. The degree to which the correction is caprurable by this adjustment is examined further in the S1 Appendix.

### Sampling uncertainty

Confidence intervals for $\hat{\lambda }$ may be constructed via bootstrap resampling [15]. If the sampling design is a simple random sample, then random with replacement samples may be used as bootstrap replicates. For more complex sampling designs, a number of resampling methods have been developed [16–19]. For the application presented in this paper, which involves the analysis of complex sample survey data, we used the multistage rescaled bootstrap method [18].

### The Kenya AIDS indicator survey (KAIS)

In 2007 and 2012, nationally representative surveys were conducted in Kenya to help inform policy makers in their HIV planning and monitoring efforts. Both studies use two-stage stratified cluster sampling. In the first stage, clusters were randomly selected from a national sampling frame (The National Sample Survey and Evaluation Programme), and in the second stage, 25 households were randomly selected within each first-stage cluster. Participants gave written informed consent to participate in the surveys and the surveys were approved by the Ethical Review Committee of the Kenya Medical Research Institute and the Institutional Review Board of the CDC. The 2012 KAIS was also approved by the Committee on Human Research of the University of California, San Francisco.

A detailed description of the sample and methods of these surveys are available in the final reports [20, 21]. A total of 17,940 individuals were included in the 2007 survey, of which 15,854 had valid serological data; 15,480 had non-missing self-reported testing history data, either reporting the time of last test, or that they had never been tested. Participants were asked: “Have you ever been tested to see if you have the virus that causes AIDS?’. If they responded “yes” they were asked: “When was the last time you were tested?”. Time since last test was categorized as less than 12 months ago, 12–23 months ago, and 2 or more years ago. ART biomarkers were collected in a subset of 578 of the 1,104 HIV positive subjects.

In the 2012 survey, 13,370 individuals were included, with 11,626 having valid serological data; 11,519 individuals had valid reported testing history information. Participants were asked: “HIV you ever been tested for HIV?” If they responded “yes”, they were asked: “When was their last HIV test (less than 3 months, 3–5 months, 6–11 months, 1–2 years, more than 2 years ago). Last test was categorized as 0–3, 3–5, 6–11, 12–24, and >24 months. ART biomarkers were collected on 559 HIV positive cases, and viral load was collected 617 of the 648 HIV positive subjects.

Assay-derived incidence was estimated using a recent infection testing algorithm (RITA) on stored specimens. In KAIS 2007, recent infection was defined as testing recent on the Limiting Antigen (LAg) Avidity Enzyme Immunoassay and having no evidence of antiretroviral therapy (ART) use. Viral load results were not available for KAIS 2007 specimens. In 2012, recent infection was defined as testing recent on LAg with HIV-1 RNA concentration at 1,000 copies/mL or higher and no ART use. For both surveys, ART use was defined as presence of the ART biomarker or self-reported ART use where ARV biomarker results were missing. For both surveys, calculation of annualized assay-derived incidence used a consensus formula for estimation of HIV incidence which incorporated a locally-derived false-recent rate derived directly estimated from a sample of known long-term infections in each survey[22]. In KAIS 2007, the FRR for the applied RITA of LAg + ART status was 1.1% (95% CI 0.3, 2.0) and in KAIS 2012, the FRR for the applied RITA of LAg + viral load status + ART status was 0.8% (95% CI 0, 2.1).

We applied our new method to generate overall incidence estimates. We also applied the method to sub-populations based on gender and geographical location. As only a small percentage of the total sample tested positive for ART biomarkers, we assumed that the rate of truthful reporting of diagnosis status (*P*(*S*|*U*^*C*^)) and the rate of positive biomarker status among the diagnosed population (*P*(*V*|*U*^*C*^)) was uniform across subgroups. This was done after an exploratory analysis determined that there was little cross group variation in these metrics.

## Results

### KAIS 2007

Table 1 shows the incidence results for both the assay-derived and testing history derived approaches. Assay-based incidence point estimates did not differ appreciably by sex. The assay-based incidence estimates are higher than the testing history-based incidence estimates.

**Table 1:** Incidence results for KAIS 2007.

	Assay Incidence % (SE)	Testing Incidence % (SE)	Testing Transmission Rate *τ* % (SE)	$\hat{P}(U|H)$	$\hat{P}(H)$	$\hat{E}(TID)$
**All**	1.10 (0.19)	0.66 (0.05)	8.65 (0.60)	0.62	0.07	7.17
**Male**	1.01 (0.25)	0.47 (0.05)	8.25 (0.64)	0.66	0.05	8.01
**Female**	1.16 (0.26)	0.84 (0.08)	9.21 (0.74)	0.60	0.08	6.52

The standard errors of the assay-based method are large, even though the number of individuals surveyed was quite large. The incidence based on testing history is more in line with expectations and consistent with the results from 2012 (see below). The bootstrap standard errors are small even in the subsets of the population.

Table 2 shows the testing history results broken down by province. Assay-derived incidence in KAIS was powered to provide estimates of national HIV incidence. Thus, the sub-national incidence estimates presented in this analysis are not expected to be reliable. The assay and testing incidence results show similar results across provinces (correlation = 0.77), with the notable exception of the Rift Valley, where the assay estimate is much higher than the testing history approach. Given that the observed prevalence of HIV was lower in 2012 than 2007, the lower incidence rate is consistent with the prevalence trends over time.

**Table 2:** Incidence results for KAIS 2007 by province.

Province	*n*	Assay Incidence % (SE)	Testing Incidence % (SE)	Testing Transmission Rate % (SE)	$\hat{P}(U|H)$	$\hat{P}(H)$	$\hat{E}(TID)$
**Central**	2277	0.65 (0.34)	0.24 (0.06)	6.41 (1.37)	0.46	0.04	7.17
**Coast**	1773	1.01 (0.57)	1.06 (0.14)	11.97 (0.98)	0.80	0.08	6.65
**Eastern**	2553	0.41 (0.26)	0.44 (0.06)	9.28 (0.93)	0.73	0.05	7.89
**Nairobi**	1811	1.58 (0.64)	1.14 (0.20)	11.77 (1.82)	0.59	0.09	4.99
**North Eastern**	753	0.17 (0.42)	0.05 (0.03)	6.39 (4.15)	0.62	0.01	9.74
**Nyanza**	2380	1.76 (0.62)	1.31 (0.17)	7.48 (0.82)	0.53	0.15	7.07
**Rift Valley**	2268	1.87 (0.45)	0.65 (0.09)	9.64 (0.94)	0.72	0.06	7.47
**Western**	2038	0.32 (0.29)	0.45 (0.07)	7.87 (1.07)	0.58	0.05	7.38

### KAIS 2012

Table 3 shows the results for 2012. Similar to 2007 the sampling variability in the testing history estimates was considerably lower than in the assay-based method. There is good agreement between the two measures in the total population estimate as well as among men and women.

**Table 3:** Incidence results for KAIS 2012.

	Assay Incidence % (SE)	Testing Incidence % (SE)	Testing Transmission Rate % (SE)	$\hat{P}(U|H)$	$\hat{P}(H)$	$\hat{E}(TID)$
**All**	0.56 (0.17)	0.50 (0.05)	8.41 (0.74)	0.33	0.06	3.96
**Male**	0.54 (0.20)	0.39 (0.05)	8.59 (0.98)	0.41	0.04	4.81
**Female**	0.58 (0.24)	0.67 (0.08)	9.14 (0.96)	0.28	0.07	3.10

Table 4 shows the testing history results broken down by province. As with the 2007 survey, the study was not designed to provide high enough sample sizes to reliably estimate provincial incidence using the assay-based approach. The assay-based incidence point estimates are high in the Eastern and Western provinces, which are low HIV burden areas [20, 21]. On the other hand, the point estimate is relatively low in Nyanza (relative to the testing history based estimate), which is a high burden area. Due to the fact that the assay-based standard errors are nearly as large as the point estimates themselves, no conclusions should be drawn from these reported patterns.

**Table 4:** Incidence results for KAIS 2012 by province (Northeastern province excluded from the sample frame due to regional insecurity).

Province	n	Assay Incidence % (SE)	Testing Incidence % (SE)	Testing Transmission Rate % (SE)	$\hat{P}(U|H)$	$\hat{P}(H)$	$\hat{E}(TID)$
Central	1423	0.16 (0.25)	0.28 (0.09)	7.11 (2.02)	0.30	0.04	4.20
Coast	1462	0.63 (0.49)	0.50 (0.12)	11.1 (2.03)	0.42	0.04	3.86
Eastern	2321	1.30 (0.51)	0.19 (0.08)	4.77 (2.02)	0.21	0.04	4.33
Nairobi	1314	0.26 (0.34)	0.70 (0.20)	13.67 (3.55)	0.36	0.05	2.46
Nyanza	1631	0.66 (0.56)	2.00 (0.31)	11.25 (1.45)	0.36	0.15	3.19
Rift Valley	2067	0.20 (0.18)	0.26 (0.07)	6.81 (1.76)	0.30	0.04	4.43
Western	1408	0.99 (0.53)	0.41 (0.13)	8.61 (2.07)	0.38	0.05	4.44

The large error bounds for the assay based estimates both in 2007 and 2012 indicate that no conclusions can be drawn regarding whether the observed decrease in incidence was due to random chance. Comparing the testing history incidence from 2007 to 2012 does show a significant drop in incidence.

## Discussion

HIV incidence is an important indicator to monitor the rate of new infection in a population to evaluate the impact of the public health response on the HIV epidemic. Increasingly, many countries rely on laboratory-based approaches to estimate national HIV incidence by measuring immunological markers of recent infection using HIV incidence assays in large populations-based HIV surveys. However, assay-derived HIV incidence estimation using cross-sectional data is challenging and limited. Large sample sizes are required for generating robust estimates of national HIV incidence using a laboratory assay and most population-based surveys are not designed to provide sub-national estimates of HIV incidence. The KAIS 2007 and KAIS 2012 surveys were not powered to estimate sub-national incidence, as reflected by large standard errors in the assay-derived estimates presented.

Alternative methods for estimating national and sub-national HIV incidence, such as model-based incidence estimation, offer an important and cost-effective option for generating HIV incidence estimates. The method proposed here has the advantage that it can be implemented using existing survey data as well as data collected from future surveys. The data requirements are attainable with little to no additional expenditure. HIV status, diagnosis history, and testing history are inexpensive to collect and commonly available in HIV prevalence surveys.

Our analysis found that the testing history-based method produced HIV incidence estimates at the national and sub-national level with acceptable levels of uncertainty. These estimates were lower than assay-derived estimates of incidence in 2007 but similar to the estimates in 2012. Though levels of HIV incidence in 2007 were expected to be higher than 2012 due to lower treatment coverage, assay-derived incidence may have been overestimated in KAIS 2007. This is due to the unavailability of viral load results to correct additional false-recent classifications on the assay that the ART biomarker did not correct for [23]. While assay-derived incidence served as a comparator against the testing history incidence estimation method, we recognize the limitations of such a comparison given that no conclusion can be made in sub-national incidence or sub-group analyses in the assay-derived estimates for KAIS due to the high level of uncertainty.

In order to obtain an incidence estimate with such minimal data requirements, it was necessary to impose some strong assumptions. Firstly, we assumed that the disease was at a steady state. If incidence had been increasing, our estimate would be low, and if it had been decreasing it would be high. Secondly, we assumed that individuals’ testing behavior is unrelated to HIV risk, and that “non-testers” would be diagnosed at progression to AIDS. Violating this assumption would bias the estimate; however, if the estimate of *E*(*TID*) is off by a percentage due to violation of this assumption, the incidence estimate may still be used to investigate trends across time and compare sub-populations. Thirdly, we assumed that all individuals had been in the population for a long time. This is violated when individuals debut into a population and may be a larger source of bias when estimating incidence in young age groups. Lastly, in constructing our adjustment to *P*(*U*|*H*), which accounts for miss-reporting, we made several simplifying assumptions. The most important of these is that biomarker-positive individuals miss-report their diagnosed status at the same rate as non-biomarker positive infected individuals. This assumption is somewhat questionable as treated individuals, who will likely have positive biomarkers, may be more accepting of their status and thus, more likely to correctly report it. In this case, the underreporting in *P*(*U*|*H*) would be only partially corrected for. However, if locally valid estimates of the differential underreporting of treated versus untreated individuals was available, this remaining bias might be removed.

Evaluating the effect of these assumptions is an open area of work. Simulation studies could improve our understanding of how sensitive the estimates are to violations of the various assumptions and the degree to which these biases could be present in hypothetical populations. Interestingly, despite the strong assumptions, our estimates were quite reasonable in both the 2007 and 2012 KAIS surveys and offer an alternative method for estimating population-based incidence. To make best use of the method, we recommend that either viral load or ART biomarker data be collected on at least a subset of individuals. This allows adjustment for misreporting of diagnosis history.

The methodology developed here has been made available as an R package downloadable from https://github.com/fellstat/TestingHistoryIncidence. Additionally, a graphical user interface for computing estimates may be accessed at https://epiapps.com/.

## Supporting information

S1 Appendix (DOCX)
