# Supplementary material for: A new method for estimating HIV incidence from a single cross-sectional survey
Source: PLoS One. 2020 Aug 12;15(8):e0237221. doi: 10.1371/journal.pone.0237221 (PMC7423136; doi:10.1371/journal.pone.0237221)
Supplement: S1 Appendix — (DOCX) [file pone.0237221.s001.docx]

**S1 Appendix. The Effect of Varying Rates of Infection**

The key assumption in deriving Equations 1 and 2 is that the infection rate (*r*) is constant over the period of the TID distribution. It is of interest to examine the behavior of the equation for r under the steady state assumption ($r=\frac{P(U|H)P(H)}{E(TID)}$) compared to its true value when infection rates are changing.

To do this we constructed a hypothetical population of 1 million individuals and tracked a disease over 60 years. The number of individuals infected each month follows a normal curve with a mean of 40 years and a standard deviation of 30 years. This allows us to examine the behavior under both increasing and decreasing infection rates as well as the inflection point between them. The curve was scaled such that at its maximum 1000 individuals are being infected each month. At the end of the 60 years, 59% of the population has been infected. For simplicity no individuals are added or removed from the population. The TID distribution is chosen to be exponential with a mean of four years. This simulation frame is designed to pinpoint the effects of varying infection rates and is not designed to match the HIV infection dynamics of any particular country, which would potentially add in additional complicating factors.

Figure 1 shows the result of this sensitivity experiment. The steady state approximation matches the true infection rate four years prior (the expected lag time of the TID distribution) very well during the increasing, decreasing regimes as well as during the inflection point. It underestimates the infection rate during the first eight years of the epidemic due to left truncation (i.e. no one was infected prior to time 0).

Figure 1: A comparison of the true infection rate with the infection rate using the steady state formula lagged by 4 years in a hypothetical disease epidemic with time varying infection rates.

**Differential miss-reporting based on biomarker status**

The methodology used to adjust for underreporting of HIV past HIV diagnosis leveraged the rate at which biomarker positive individuals incorrectly classified themselves as undiagnosed. In equation 5 of the paper we assumed that this miss-reporting rate was the same in non-biomarker positive individuals with previous HIV diagnoses.

If this assumption does not hold, it is reasonable to assume that the non-biomarker positives individuals miss-report at a higher rate compared to biomarker positive individuals (1). The result of this would be that the estimate of P(U|H) would remain inflated, though to a lesser extent than the unadjusted estimate.

In evaluating the effect of differential miss-reporting it is useful to consider the probability of being diagnosed

$$P\left( U^{C} | H \right)=\frac{P(S\cup V|H)}{P(S\cup V|U^{c})}.$$

This corrects the naive proportion of self-reported / biomarker confirmed by an adjustment factor $\frac{1}{P(S\cup V|U^{c})}$. By assuming *S* and *V* are independent, our estimator replaces this adjustment factor with one that is estimable from the data

$$\frac{1}{P(S|V)+P(V|S)(1-P(S|V))}.$$

We explored the degree to which the estimable adjustment factor differs from the true adjustment factor when half of the population has the biomarker ($P\left( V \right)=0.5$) and the rate of miss-reporting in the non-biomarker group is 30% ($P\left( S | V^{C} \right)= .7$). We varied the reporting rate in the biomarker group compared to the non-biomarker group from an odds ratio of 1 to 1.5.

| Odds Ratio | Estimable Adjustment | True Adjustment |
| --- | --- | --- |
| 1.0 | +18% | +18% |
| 1.1 | +16% | +18% |
| 1.2 | +15% | +18% |
| 1.3 | +14% | +18% |
| 1.4 | +13% | +18% |
| 1.5 | +12% | +18% |

Table 1: Comparison of true vs estimable adjustment factors

Table 1 compares the estimable adjustment factor to its true value. We see that both the true and estimable version are equal to one another when there is no differential miss-reporting (odds ratio = 1). As the differential reporting increases, so does the deviation from the true value. At the most extreme level, the estimable adjustment value scales up by 12% whereas it should have scaled up by 18%.

If this odds ratio were known via an alternative estimate, the estimable adjustment factor could be corrected to account for differential miss-reporting. This is an area for future research.

**References**

1. Fuente‐Soro, L., Lopez‐Varela, E., Augusto, O., Sacoor, C., Nhacolo, A., Honwana, N., ... & Naniche, D. (2018). Monitoring progress towards the first UNAIDS target: understanding the impact of people living with HIV who re‐test during HIV‐testing campaigns in rural Mozambique. *Journal of the International AIDS Society*, *21*(4), e25095.
